# Current diagnosis and management of Crohn’s disease in China: results from a multicenter prospective disease registry

**DOI:** 10.1186/s12876-019-1057-2

**Published:** 2019-08-16

**Authors:** Yue Li, Baili Chen, Xiang Gao, Naizhong Hu, Meifang Huang, Zhihua Ran, Zhanju Liu, Jie Zhong, Duowu Zou, Xiaoping Wu, Jianlin Ren, Jianqiu Sheng, Ping Zheng, Huahong Wang, Minhu Chen, Junrong Chen, Peng Xi, Jiajia Lu, Malcolm Handel, Yanfang Liu, Hua Fan, Jiaming Qian

**Affiliations:** 1 Peking Union Medical College Hospital, Chinese Academy of Medical Sciences and Peking Union Medical College, Beijing, China; 2 The First Affiliated Hospital, Sun Yat-sen University, Guangzhou, China; 3 The Sixth Affiliated Hospital, Sun Yat-sen University, Guangzhou, China; 4 The First Affiliated Hospital of Anhui Medical University, Hefei, China; 5 Zhongnan Hospital of Wu Han University, Wuhan, China; 6 Renji Hospital, Shanghai Jiao Tong University School of Medicine, Shanghai, China; 7 Shanghai Tenth Peoples Hospital of Tongji University, Shanghai, China; 8 Ruijin Hospital, Shanghai Jiao Tong University School of Medicine, Shanghai, China; 9 Changhai Hospital, Second Military Medical University, Shanghai, China; 10 The Second Xiangya Hospital of Central South University, Changsha, China; 11 Zhongshan Hospital, Xiamen University, Xiamen, China; 12 Army General Hospital, Beijing, China; 13 Dongfang Hospital of Tongji University, Shanghai, China; 14 Peking University First Hospital, Beijing, China; 15 Medical Affairs, Xi’an Janssen Pharmaceutical Ltd., Beijing, China; 16 Johnson & Johnson (China) Investment Ltd. Janssen China R&D Center, Beijing, China; 17 Janssen Asia-Pacific, Sydney, Australia; 18 Janssen Asia-Pacific, Singapore, Singapore; 19 Medical Affairs, Takeda China, Shanghai, China

**Keywords:** China, Crohn’s disease, Disease registry, Extraintestinal manifestations, Treatment outcomes

## Abstract

**Background:**

This study aimed to understand the disease characteristics and treatment outcomes of Crohn’s disease (CD) in a real-world setting in China.

**Methods:**

In this prospective, non-interventional, multicenter disease registry, adults (≥18 years) with existing and newly diagnosed CD were recruited from 14 medical centers across China from January 2015 to January 2017. The study consisted of the enrollment and follow-up periods, of 12 months each. Demographic, clinical characteristics, diagnostic duration and management of CD at enrollment were evaluated. Logistic regression analysis and stepwise multivariate logistic regression analysis used to assess the relationship between the risk factors and CD.

**Results:**

Of 504 enrolled patients, 499 (99.0%) were eligible for analysis. The mean (SD) age at study enrollment was 32.3 (11.43) years and the majority (69.7%) of participants were male. In the past 15 years, a sustained decrease of the period of time in the diagnosis of CD was observed, at about 39.4 (24.11) months in 2010, which decreased to 3.1 (2.13) months in 2015. The most common presenting symptoms of CD included abdominal pain (78.0%), diarrhea (58.1%), weight loss (52.9%) and fever (30.1%). Oral ulcer (19.4%) and arthritis (9.8%) were the most common extra-intestinal manifestations. Non-stricturing non-penetrating (B1) (49.9%) behavior and ileocolonic involvement (L3) (56.2%) location were more frequent. Perianal disease was observed in 29.1% of the patients. Around 23.8% (119/499) patients had CD-related surgery other than perianal disease surgery. Older age at enrollment, longer disease course, complicated disease behavior and absence of perianal disease were all surgery risk factors (*p* < 0.05). The most common medications was immunomodulators (e.g., azathioprine) (41.5%), anti-TNFα agents (32.9%) and aminosalicylates (20.6%). The mean (SD) Crohn’s Disease Active Index (CDAI) score was 159.1 (91.45) and almost half of the patients (49.1%, 81/165) were in remission.

**Conclusions:**

This study demonstrated the CD-disease characteristics, risk factors of CD-related surgery and perianal disease, and treatment strategies in a real-world setting in China and may help in developing programs to diagnose and manage patients with CD.

**Electronic supplementary material:**

The online version of this article (10.1186/s12876-019-1057-2) contains supplementary material, which is available to authorized users.

## Background

Crohn’s disease (CD) is a chronic, progressive and relapsing inflammatory bowel disease (IBD) that is often characterized by the presence of intestinal strictures, fistulas or abscesses [1]. The incidence rate of CD in Western countries has demonstrated a significant increasing trend, and it varies from 0.3 to 12.7 cases per 100,000 persons in Europe and 0 to 20.2 cases per 100,000 persons in North America [2, 3]. Similar to Western countries, the incidence of CD has increased over time in Asian countries [4–7]. While CD is well known in Western countries, it was reported in China about 30 years later: the first case of CD was reported in China in 1956 [8]. Although the overall incidence of CD in China is much lower than in Western countries, the incidence is increasing rapidly with urbanization and socioeconomic development [9]. The current estimated incidence is 0.51–1.09 cases per 100,000 persons in China [10, 11]. In Hong Kong, the incidence of CD has tripled over the last 10 years [12]. Since most studies focused only on particular regions of China, existing data are inadequate to represent the actual incidence of CD at the national level.

According to available data on CD, there is a substantial difference in the incidence and disease characteristics of CD in southern and northern China, as well as in coastal and inland areas [13, 14]. China is also known as a high-risk area for infectious diseases, especially tuberculosis [15]. Due to similar clinical, morphological and histological features of intestinal tuberculosis and CD, the differential diagnosis between these two disorders remains challenging, and must also be considered in terms of treatment modalities and strategies. Intriguingly, while the epidemiology, phenotypic characteristics and management of CD in Asia have the same features as that of Western countries, they also possess certain specific characteristics. A comprehensive understanding of the demographics and disease characteristics as well as diagnosis and treatment status of patients with CD is of great significance to improve the disease management in China. Furthermore, understanding of these factors can likely help optimize diagnosis and treatment and provide the basis for making public health policy decisions.

There is a paucity of population-based epidemiological studies to demonstrate the disease status, diagnosis and treatment patterns of CD in China. The aim of the present study was to build a hospital-based CD registry that would help to analyze the disease characteristics, diagnosis and treatment patterns, and potentially support understanding of treatment strategies for CD in clinical practice in China. Results of this study would help optimize and develop management strategies based on characteristics of CD patients in China.

## Methods

### Patients

Patients aged ≥18 years were recruited from 14 tertiary teaching medical centers in China from January 2015 to January 2017 (Additional file 1: Table S1). Patients were included if they had a confirmed diagnosis of CD, attended the outpatient clinics and/or hospital wards and were adhering to the physician’s advice through follow-up for up to 1 year.

### Study design

In this prospective, non-interventional, multicenter disease registry, patients with a confirmed diagnosis of CD were enrolled regardless of the treatments received. All the eligible patients were followed for 1 year.

### Study objectives

The primary objectives of the study were to collect data related to patient demographics and clinical manifestations. Secondary objectives included risk factors for different disease behaviors and locations, and factors related to surgery and medical treatment.

### Study evaluations

At baseline, data were collected for variables including patient demographics, nicotine use history, alcohol use history, medical history and comorbid medical conditions, disease onset and diagnosis time, CD symptom profile, details of prior treatment regimen and surgery history. Surgery history included CD-related surgery and perianal disease-related surgery. CD-related surgery referred to any bowel resection due to CD complications. Baseline data were collected upon entry into the registry. During the observational period, each patient was followed for 1 year and data were collected approximately every 3 months to observe their clinical progression.

Patients were grouped based on location of the medical centers as coastal cities: Shanghai, Hangzhou, Guangzhou, Xiamen, and inland cities: Beijing, Changsha, Wuhan, Hefei (Additional file 1: Table S1). The Montreal classification was used to define disease location as L1: terminal ileum, L2: colon, L3: ileocolon and L4: involving upper gastrointestinal tract. Disease behavior was defined as B1: non-stricturing non-penetrating, B2: stricturing and B3: penetrating [16]. The disease activity was assessed using the Crohn’s Disease Active Index (CDAI).

Demographics and disease characteristics such as age, sex, body mass index, nicotine and alcohol use, family and medical history, months from disease onset to enrollment and months from initial definite diagnosis to enrollment were correlated at baseline.

### Statistical analysis

A sample size of approximately 500 patients from 14 tertiary medical centers was needed to obtain data that was representative of CD patients in China. Eligible patients were enrolled in the registry irrespective of their treatments, and as this was a descriptive study, no formal hypotheses were tested in this registry. The sample size was determined by pragmatic considerations including feasibility and cost. Effectiveness outcomes were based on the full analysis set (FAS), which included patients who entered the registry and had at least one assessment visit during the treatment period. Baseline data were presented and analyzed in this study. All continuous variables were summarized using mean (standard deviation [SD]). All categorical variables were summarized using frequencies and percentages. Logistic regression analysis and a stepwise multivariate logistic regression analysis was conducted to examine the relationship between the risk factors and CD with entry α = 0.5 and stay α = 0.2 as the selection criteria. The 95% confidence interval (CI) and odds ratio (OR) were calculated. Statistically significant (*p* < 0.05) results obtained from this analysis of correlation factors using logistic regression model are presented here.

### Ethical considerations

This study was conducted in accordance with the ethical principles of the Declaration of Helsinki and the International Conference on Harmonization Good Clinical Practice guidelines. The Independent Ethics Committee or the Institutional Review Board at each study site approved the trial design and eligible patients provided written informed consent before participating.

## Results

### Enrollment and demographic characteristics

During the study period, 504 patients were screened and 499 patients were eligible. Among those 499 patients, 421 (84.4%) completed the registry and 1-year observational phase; 78 patients were excluded from the analysis mainly due to loss to follow-up (*n* = 60) and withdrawal of consent (*n* = 12) (Additional file 1: Table S2). Among 499 patients, 32.9% (164/499) were from South China, 28.9% (144/499) from East China, 21.1% (106/499) from Central China and 17.0% (85/499) from North China (Additional file 1: Table S1). About 61.7% (308/499) of patients were enrolled in centers located in coastal cities, and 38.3% (191/499) enrolled in centers in inland cities. The mean (SD) age was 32.3 (11.43) years (median: 30.0, range: 18 to 77 years), and a higher proportion of patients were male (69.7%, *n* = 348, male vs. female ratio: 2.30:1). The majority of patients never used nicotine (86.4%, *n* = 382) and never consumed alcohol (90.5%, *n* = 401). Patents enrolled in inland medical centers had a higher proportion of smoking (current user 10.3% vs. 6.9%, former user 9.7% vs. 2.9%, *p* = 0.0036) and drinking (former user 9.0% vs. 1.8%, *p* = 0.0023) than that of patients enrolled in coastal cities (Table 1). At baseline, hepatitis B was reported in 19 (3.8%) patients. Appendicitis and medical history of nephrolithiasis were reported in 10 (2.0%) patients each (Table 1).

**Table 1:** Demographic and clinical characteristics of patients with Crohn’s disease at baseline

Variable	Total	Inland city	Coastal city	*P* value
N (%)	499	191 (38.3%)	308 (61.7%)	
Age at enrollment (years), mean (SD)	32.3 (11.43)	34.3 (13.03)	31.1 (10.14)	0.0022
Male, n (%)	348 (69.7%)	138 (72.3%)	210 (68.2%)	
Ethnicity, n (%)				
Chinese Han	495 (99.2%)	187 (97.9%)	308 (100.0%)	
Other Chinese minority	4 (0.8%)	4 (2.1%)	0	
BMI (kg/m^2^), mean (SD)	19.10 (3.198)	18.80 (2.676)	19.29 (3.479)	
Nicotine use history, n (%)				0.0036
N	442	165	277	
Never used	382 (86.4%)	132 (80.0%)	250 (90.3%)	
Current user	36 (8.1%)	17 (10.3%)	19 (6.9%)	
Former user	24 (5.4%)	16 (9.7%)	8 (2.9%)	
Alcohol use history, n (%)				0.0023
N	443	166	277	
Never used	401 (90.5%)	143 (86.1%)	258 (93.1%)	
Current user	22 (5.0%)	8 (4.8%)	14 (5.1%)	
Former user	20 (4.5%)	15 (9.0%)	5 (1.8%)	
Medical history reported, n (%)				
Any medical history	180 (36.1%)	76 (39.8%)	104 (33.8%)	
Hepatitis B	19 (3.8%)	7 (3.7%)	12 (3.9%)	
Appendicitis	10 (2.0%)	5 (2.6%)	5 (1.6%)	
Hypertension	8 (1.6%)	3 (1.6%)	5 (1.6%)	
Nephrolithiasis	10 (2.0%)	7 (3.7%)	3 (1.0%)	
CD-related surgery history, n (%)	119 (23.8%)	51 (26.7%)	68 (22.1%)	0.2071
Perianal disease related surgery, n (%)	79 (15.8%)	21 (11.0%)	58 (18.8%)	0.0023
Family history of IBD, n (%)				
N	6	2	4	
Crohn’s disease	5 (83.3%)	2 (100%)	3 (75.0%)	
Ulcerative colitis	1 (16.7%)	0	1 (25.0%)	
Age at disease onset (years), n (%)				0.0291
≤ 16	29 (5.8%)	15 (7.9%)	14 (4.5%)	
17–39	401 (80.4%)	142 (74.3%)	259 (84.1%)	
≥ 40	69 (13.8%)	34 (17.8%)	35 (11.4%)	
Age at initial diagnosis (years), n (%)				0.0083
≤ 16	6 (1.2%)	4 (2.1%)	2 (0.6%)	
17–39	418 (83.8%)	148 (77.5%)	270 (87.7%)	
≥ 40	75 (15.0%)	39 (20.4%)	36 (11.7%)	
Time from disease onset to initial diagnosis (months), mean (SD)	32.0 (46.43)	39.9 (58.84)	27.0 (35.93)	0.0255
Location, n (%)				< 0.0001
N	493	190	303	
L1	137 (27.8%)	69 (36.3%)	68 (22.4%)	
L2	71 (14.4%)	38 (20.0%)	33 (10.9%)	
L3	277 (56.2%)	82 (43.2%)	195 (64.4%)	
L4	8 (1.6%)	1 (0.5%)	7 (2.3%)	
Behavior, n (%)				< 0.0001
N	495	190	305	
B1	247 (49.9%)	110 (57.9%)	137 (44.9%)	
B2	148 (29.9%)	45 (23.7%)	103 (33.8%)	
B3	100 (20.2%)	35 (18.4%)	65 (21.3%)	
Perianal disease	144 (29.1%)	32 (16.8%)	112 (36.7%)	
Crohn’s Disease Activity Index at baseline, n (%)				0.0094
N	165	80	85	
< 150	81 (49.1%)	37 (46.3%)	44 (51.8%)	
150–220	43 (26.1%)	28 (35.0%)	15 (17.6%)	
221–400	39 (23.6%)	13 (16.3%)	26 (30.6%)	
> 400	2 (1.2%)	2 (2.5%)	0	
Crohn’s disease treatment information at baseline, n (%)				
Any Crohn’s disease treatment	441 (88.4%)	161 (84.3%)	280 (90.9%)	0.0308
Immunomodulators	207 (41.5%)	78 (40.8%)	129 (41.9%)	0.8179
Anti-TNF agents	164 (32.9%)	50 (26.2%)	114 (37.0%)	0.0123
Aminosalicylates	103 (20.6%)	47 (24.6%)	56 (18.2%)	0.0847
Steroids	99 (19.8%)	53 (27.7%)	46 (14.9%)	0.0005
Enteral nutrition	69 (13.8%)	6 (3.1%)	63 (20.5%)	< 0.0001
Antibiotics	65 (13.0%)	6 (3.1%)	59 (19.2%)	< 0.0001
Anti-tuberculosis	27 (5.4%)	1 (0.5%)	26 (8.4%)	< 0.0001
Anti-hepatitis	3 (0.6%)	2 (1.0%)	1 (0.3%)	0.5613
Other agents^a^	115 (23.0%)	7 (3.7%)	108 (35.1%)	< 0.0001

*B1* non-stricturing non-penetrating, *B2* stricturing, *B3* penetrating, *BMI* body mass index, *CD* Crohn’s disease, *IBD* inflammatory bowel disease, *L1* terminal ileum, *L2* colon, *L3* ileocolon, *L4* upper gastrointestinal tract, *n* total number of participants in a subset, *N* total sample size, *SD* standard deviation, *TNF* tumor necrosis factor

^a^Other agents including traditional Chinese medicine, et al

**Fig. 1:**
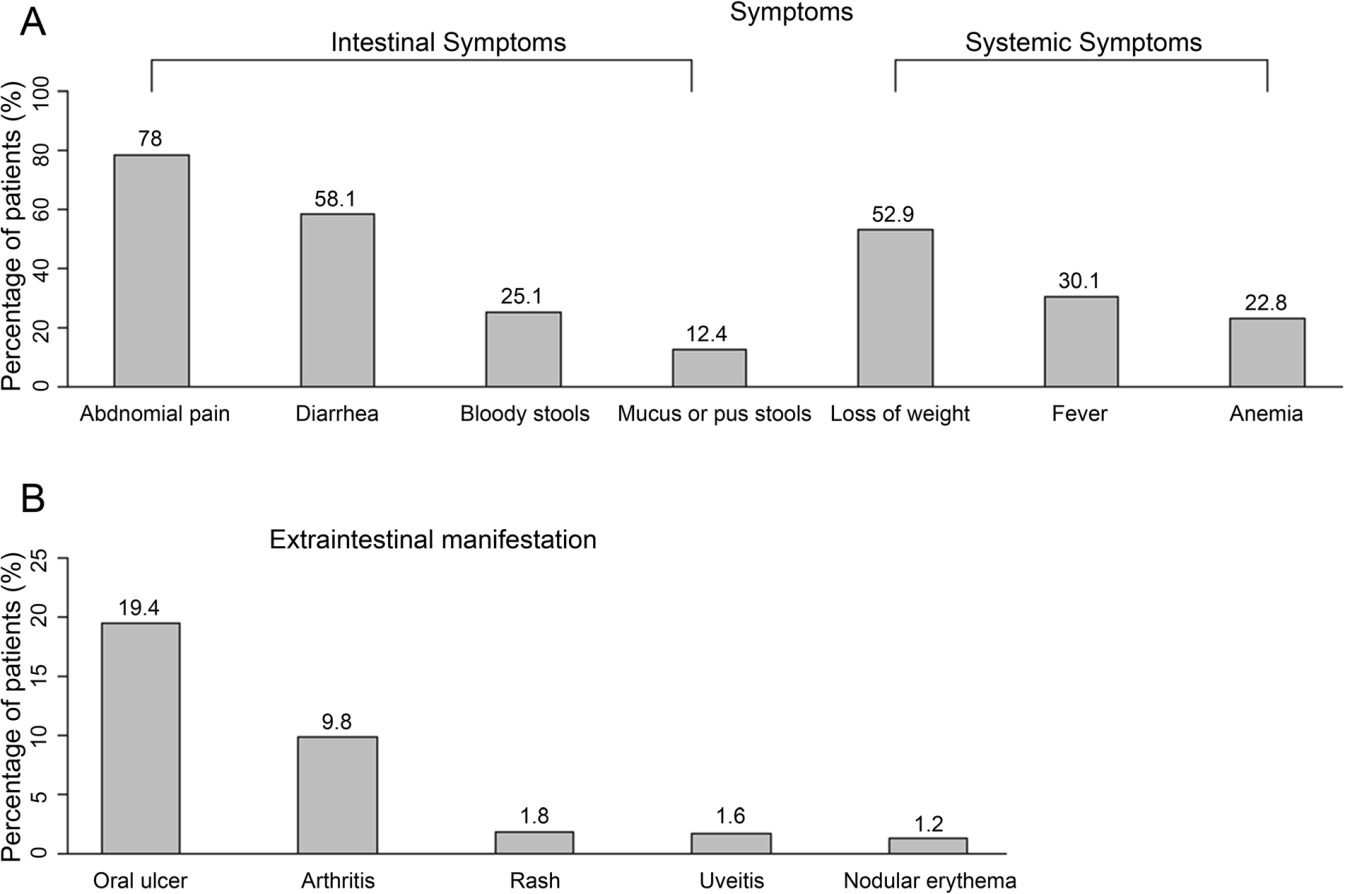
Symptom and complication profile (**a**) and extraintestinal manifestations (**b**) in patients with Crohn’s disease

Abdominal pain (78.0%, 389/499), diarrhea (58.1%, 290/499), weight loss (52.9%, 264/499) and fever (30.1%, 150/499) were the most common symptoms of CD. Less common symptoms were bloody stool (25.1%, 125/499), anemia (22.8%, 114/499) and mucus or pus in stools (12.4%, 62/499) (Fig. 1a). Oral ulcer (19.4%, 97/499) and arthritis (9.8%, 49/499) were the most common extra-intestinal manifestations. Skin lesions such as nodular erythema and uveitis were relatively uncommon (Fig. 1b).

### Time from disease onset to definite diagnosis

**Fig. 2:**
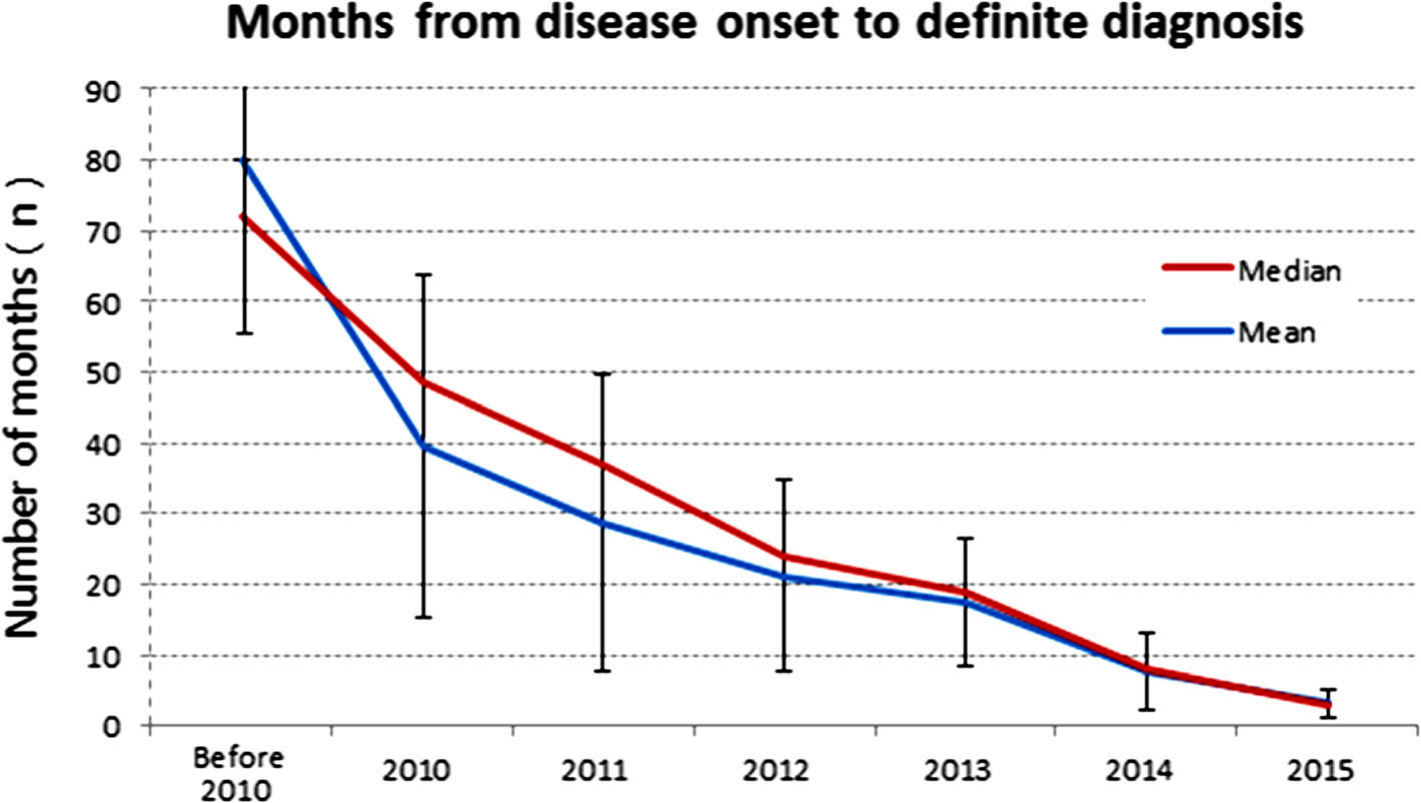
Months from disease onset to definite diagnosis

The mean (SD) time from disease onset to definite diagnosis was 32.0 (46.43) months, patients enrolled in inland centers had a longer duration for diagnosis (39.9 [58.84] vs. 27.0 [35.93] months, *p* = 0.0255) than patients enrolled in coastal centers. The patients with data available for different time periods were grouped by the year of disease onset (Table 2). The mean (SD) duration from disease onset to initial definitive diagnosis decreased significantly over the period of time from 79.4 (67.03) months before 2010, to 39.4 (24.11) months in 2010, and to 3.1 (2.13) months in 2015 (Table 2 and Fig. 2). There was a significant shortening of diagnostic duration in recent years.

**Table 2:** Crohn’s disease diagnosis information - grouped by duration from disease onset to date of initial definite diagnosis

Year of disease onset	n	Age (years) at disease onset^a^	Months from disease onset to definite diagnosis^b^
Before 2010	130	27.0 (10.82)	79.4 (67.03)
2010	34	28.1 (11.73)	39.4 (24.11)
2011	38	26.4 (10.65)	28.7 (20.81)
2012	41	28.6 (10.07)	21.0 (13.57)
2013	74	26.3 (8.48)	17.3 (8.99)
2014	104	28.7 (10.74)	7.7 (5.41)
2015	78	30.5 (11.53)	3.1 (2.13)

All values are mean (standard deviation) unless otherwise stated

*n* total number of participants in a subset

^a^Age at disease onset is calculated as (date of disease onset-date of birth+ 1)/365.25, rounded to integer

^b^Time from disease onset to definite diagnosis, measured by (date of definite diagnosis-date of disease onset), rounded to integer

### Surgery history

Of the total 499 patients, 23.8% (119/499) had a history of CD-related surgery, and 15.8% (19/499) had a history of perianal disease related surgery. Demographics and baseline characteristics were comparable between patients with history of CD-related surgery and patients without. Patients who had a CD-related surgery history were older at disease onset (30.8 ± 10.98 vs. 27.0 ± 10.37 years, *p* = 0.0006), at initial definite diagnosis (34.1 ± 11.45 vs. 29.5 ± 10.66 years, *p* < 0.0001), and at enrollment (37.3 ± 11.95 vs. 30.8 ± 10.81 years, *p* < 0.0001) than patients without CD-related surgery history (Table 3). Time from disease onset to initial diagnosis (39.4 ± 47.57 vs. 29.6 ± 45.89 months, *p* = 0.0457) as well as time from disease onset to enrollment (78.6 ± 73.29 vs. 44.4 ± 51.32 months, *p* < 0.0001) were significantly longer in patients with a history of CD-related surgery. Fistulizing or penetrating CD behavior (B3) was higher (43.5% vs. 13.2%, *p* < 0.0001) in patients with history of CD-related surgery than in patients without history of CD-related surgery. On the contrary, patients with a CD-related surgery history had lower presence of perianal disease (16.8%, *n* = 20 vs. 32.6%, *n* = 124, *p* = 0.0009). Logistic model for correlation analysis revealed older age at enrollment (OR = 1.047, *p* < 0.01), longer disease course (months from disease onset to enrollment, OR = 1.009, *p* < 0.01), stricturing behavior (B2 vs. B1, OR = 2.815, *p* < 0.001), penetrating behavior (B3 vs B1, OR = 8.147, *p* < 0.001; B3 vs. B2, OR = 2.895, *p* < 0.001) and perianal disease (OR = 0.419, *p* < 0.001) were associated with CD-related surgery (Table 4).

**Table 3:** Demographic and clinical characteristics - grouped by baseline surgery information

Variable	Total	Patients with surgery history	Patients without surgery history	*P* value
N (%)	499	119 (23.8%)	380 (76.2%)	
Age (years), mean (SD)	32.3 (11.43)	37.3 (11.95)	30.8 (10.81)	< 0.0001
Male, n (%)	348	91 (76.5%)	257 (67.6%)	0.0670
BMI (kg/m^2^), mean (SD)	19.10 (3.198)	18.81 (3.119)	19.19 (3.223)	0.3898
Age at disease onset (years), mean (SD)	27.9 (10.63)	30.8 (10.98)	27.0 (10.37)	0.0006
Age group at disease onset (years), n (%)				0.0007
N	499	119	380	
≤ 16	29 (5.8%)	6 (5.0%)	23 (6.1%)	
17–39	401 (80.4%)	84 (70.6%)	317 (83.4%)	
≥ 40	69 (13.8%)	29 (24.4%)	40 (10.5%)	
Age at initial definite diagnosis (years), mean (SD)	30.6 (11.01)	34.1 (11.45)	29.5 (10.66)	< 0.0001
Time from disease onset to initial diagnosis (months), mean (SD)	32.0 (46.43)	39.4 (47.57)	29.6 (45.89)	0.0457
Time from initial diagnosis to enrollment (months), mean (SD)	20.6 (39.06)	39.3 (61.42)	14.8 (26.24)	< 0.0001
Time from disease onset to enrollment (months), mean (SD)	52.6 (59.08)	78.6 (73.29)	44.4 (51.32)	< 0.0001
Nicotine use history, n (%)				0.4698
N	442	99	343	
Never used	382 (86.4%)	83 (83.8%)	299 (87.2%)	
Current user	36 (8.1%)	11 (11.1%)	25 (7.3%)	
Former user	24 (5.4%)	5 (5.1%)	19 (5.5%)	
Alcohol use history, n (%)				0.0760
N	443	99	344	
Never used	401 (90.5%)	84 (84.8%)	317 (92.2%)	
Current user	22 (5.0%)	8 (8.1%)	14 (4.1%)	
Former user	20 (4.5%)	7 (7.1%)	13 (3.8%)	
Location, n (%)				0.4628
N	493	115	378	
L1	137 (27.8%)	27 (23.5%)	110 (29.1%)	
L2	71 (14.4%)	21 (18.3%)	50 (13.2%)	
L3	277 (56.2%)	65 (56.5%)	212 (56.1%)	
L4	8 (1.6%)	2 (1.7%)	6 (1.6%)	
Behavior, n (%)				< 0.0001
N	495	115	380	
B1	247 (49.9%)	27 (23.5%)	220 (57.9%)	
B2	148 (29.9%)	38 (33.0%)	110 (28.9%)	
B3	100 (20.2%)	50 (43.5%)	50 (13.2%)	
Perianal disease, n (%)	144 (28.9%)	20 (16.8%)	124 (32.6%)	0.0009

*B1* non-stricturing non-penetrating, *B2* stricturing, *B3* penetrating, *BMI* body mass index, *L1* terminal ileum, *L2* colon, *L3* ileocolon, *L4* upper gastrointestinal tract, *SD* standard deviation

**Table 4:** Logistic model for each correlation factors to baseline CD-related surgery history (has surgery history vs. no surgery history)

Variable	OR	95% CI for OR	Parameter estimates (standard error)	Corresponding 95% CI	*P* value
Age (years)	1.047	1.029, 1.066	0.046 (0.009)+	0.029, 0.064	< 0.001*
Sex (male vs. female)	1.549	0.963, 2.491	0.438 (0.242)+	−0.037, 0.913	0.071
BMI (18.5 - < 24 vs. < 18.5 kg/m^2^)	0.845	0.482, 1.481	−0.169 (0.287)	−0.731, 0.393	0.555
BMI (≥24 vs. < 18.5 kg/m^2^)	0.713	0.225, 2.260	− 0.339 (0.589)	−1.493, 0.815	0.565
Nicotine use (former vs. Never)	0.951	0.345, 2.624	−0.050 (0.518)	−1.065, 0.965	0.923
Nicotine use (current vs. Never)	1.591	0.752, 3.367	0.464 (0.382)+	−0.285, 1.214	0.225
Alcohol use (former vs. Never)	2.038	0.789, 5.270	0.712 (0.485)+	−0.238, 1.662	0.142
Alcohol use (current vs. Never)	2.163	0.878, 5.328	0.772 (0.460)+	−0.130, 1.673	0.093
Time from disease onset to enrollment (months)	1.009	1.005, 1.013	0.009 (0.002)+	0.005, 0.012	< 0.001*
Time from initial definite diagnosis to enrollment (months)	1.015	1.009, 1.021	0.015 (0.003)+	0.009, 0.021	< 0.001*
Disease behavior (B2 vs. B1)	2.815	1.634, 4.848	1.035 (0.278)+	0.491, 1.579	< 0.001*
Disease behavior (B3 vs. B1)	8.147	4.655, 14.261	2.098 (0.286)+	1.538, 2.658	< 0.001*
Disease behavior (B3 vs. B2)	2.895	1.690, 4.959	1.063 (0.275)+	0.525, 1.601	< 0.001*
Disease location (L3 vs. L1)	1.260	0.761, 2.087	0.231 (0.257)	−0.273, 0.736	0.368
Disease location (L3 vs. L2)	0.730	0.408, 1.304	−0.315 (0.296)+	−0.895, 0.266	0.288
Perianal disease (yes vs. no)	0.419	0.247, 0.709	−0.871 (0.268)+	−1.397, − 0.344	0.001*

“No surgery history” is the reference level. +: standard error is not larger than parameter estimates. *: *P* < 0.05. *B1* non-stricturing non-penetrating, *B2* stricturing, *B3* penetrating, *BMI* body mass index, *CI* confidence interval, *L1* terminal ileum, *L2* colon, *L3* ileocolon, *OR* odds ratio

### Disease behavior and location

At baseline, non-stricturing non-penetrating (B1) disease behavior (49.9%, 247/495) was the highest. Centers in coastal cities were taking care of more patients with B2 (33.8% vs. 23.7%), B3 (21.2% vs. 18.4%) behavior and perianal disease (36.7% vs. 16.8%) than inland centers (*p* < 0.0001). Increase in duration from disease onset to enrollment and increase in duration from disease initial definite diagnosis to enrollment in registry were risk factors associated with patients of B2 and B3 behavior (OR = 1.006, *p* = 0.002; and OR = 1.007, *p* = 0.009, respectively). Currently smoking (OR = 2.454, *p* = 0.020) was also associated with patients with B2 and B3 behavior (Additional file 1: Table S3).

**Fig. 3:**
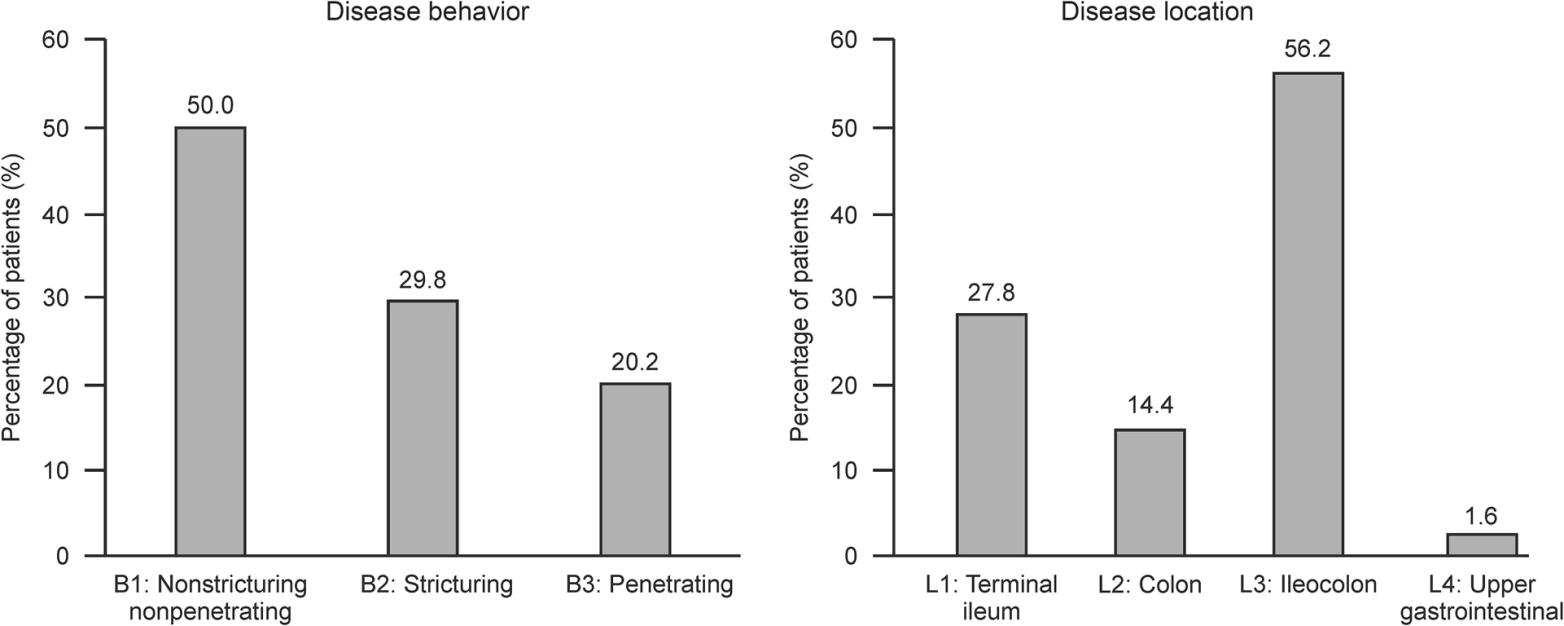
Disease behavior (**a**) and disease location (**b**) classification of patients with Crohn’s disease. B1: non-stricturing non-penetrating; B2: stricturing; B3: penetrating; L1: terminal ileum; L2: colon; L3: ileocolon; L4: upper gastrointestinal tract

CD was classified as being localized in the L3 region in the majority of patients (56.2%, 277/493) (Fig. 3). Former smokers had higher probability of having CD localized in terminal ileal (L1) region than ileocolonic (L3) region (OR = 0.205, *p* = 0.004, Additional file 1: Table S4). Body mass index (BMI) in normal range (18.5–24 kg/m^2^) was associated with colonic (L2) location compared with L1 location (OR = 0.404, *p* = 0.017).

Patients who had perianal disease were younger at disease onset (25.3 ± 9.40 vs. 29.0 ± 10.92 years, *p* = 0.0003), at initial definite diagnosis (27.7 ± 10.02 vs. 31.8 ± 11.19 years, *p* = 0.0001), and at enrollment (28.9 ± 10.05 vs. 33.7 ± 11.67 years, *p* < 0.0001) than patients without perianal disease (Table 5). Duration from initial diagnosis to enrollment (14.8 ± 24.97 vs. 22.9 ± 43.29 months, *p* = 0.0345) and duration from disease onset to enrollment (43.8 ± 44.55 vs. 56.2 ± 63.75 months, *p* = 0.0341) were significantly shorter in patients with perianal disease. Logistic model for correlation analysis revealed that younger age at enrollment (OR = 0.957, *p* < 0.001), shorter disease course (time from disease onset to enrollment, OR = 0.996, *p* = 0.036), smoking (former vs. never, OR = 0.209, *p* = 0.036), B2 disease behavior (OR = 0.514, *p* = 0.006), and L3 disease location (OR = 3.052, *p* < 0.001) were associated with perianal disease (Table 6). Patients with perianal disease had higher proportion of anti-tumor necrosis factor α (TNFα) agents use (41.7% vs. 29.6%, *p* = 0.0098) and antibiotics use (20.8% vs. 10.0%, *p* = 0.0012).

**Table 5:** Demographic and clinical characteristics - grouped by baseline perianal disease

Variable	Total	Patients with perianal disease	Patients without perianal disease	*P* value
N (%)	499	144 (28.9%)	355 (71.1%)	
Age (years), mean (SD)	32.3 (11.43)	28.9 (10.05)	33.7 (11.67)	< 0.0001
Male, n (%)	348 (69.7%)	102 (70.8%)	246 (69.3%)	0.7348
BMI (kg/m^2^), mean (SD)	19.10 (3.198)	19.29 (3.254)	19.04 (3.182)	0.5446
Age at disease onset (years), mean (SD)	27.9 (10.63)	25.3 (9.40)	29.0 (10.92)	0.0003
Age group at disease onset (years), n (%)				0.0556
N	499	144	355	
≤ 16	29 (5.8%)	7 (4.9%)	22 (6.2%)	
17–39	401 (80.4%)	125 (86.8%)	276 (77.7%)	
≥ 40	69 (13.8%)	12 (8.3%)	57 (16.1%)	
Age at initial definite diagnosis (years), mean (SD)	30.6 (11.01)	27.7 (10.02)	31.8 (11.19)	0.0001
Time from disease onset to initial diagnosis (months), mean (SD)	32.0 (46.43)	29.0 (38.54)	33.2 (49.27)	0.3672
Time from initial diagnosis to enrollment (months), mean (SD)	20.6 (39.06)	14.8 (24.97)	22.9 (43.29)	0.0345
Time from disease onset to enrollment (months), mean (SD)	52.6 (59.08)	43.8 (44.55)	56.2 (63.75)	0.0341
Nicotine use history, n (%)				0.0188
N	442	124	318	
Never used	382 (86.4%)	116 (93.5%)	266 (83.6%)	
Current user	36 (8.1%)	6 (4.8%)	30 (9.4%)	
Former user	24 (5.4%)	2 (1.6%)	22 (6.9%)	
Alcohol use history, n (%)				0.3203
N	443	124	319	
Never used	401 (90.5%)	116 (93.5%)	285 (89.3%)	
Current user	22 (5.0%)	3 (2.4%)	19 (6.0%)	
Former user	20 (4.5%)	5 (4.0%)	15 (4.7%)	
Location, n (%)				< 0.0001
N	493	143	350	
L1	137 (27.8%)	23 (16.1%)	114 (32.6%)	
L2	71 (14.4%)	14 (9.8%)	57 (16.3%)	
L3	277 (56.2%)	105 (73.4%)	172 (49.1%)	
L4	8 (1.6%)	1 (0.7%)	7 (2.0%)	
Behavior, n (%)				0.0309
N	495	144	351	
B1	247 (49.9%)	84 (58.3%)	163 (46.4%)	
B2	148 (29.9%)	32 (22.2%)	116 (33.0%)	
B3	100 (20.2%)	28 (19.4%)	72 (20.5%)	
Crohn’s disease treatment information at baseline, n (%)				
Immunomodulators	207 (41.5%)	64 (44.4%)	143 (40.7%)	0.4480
Anti-TNF agents	164 (32.9%)	60 (41.7%)	104 (29.6%)	0.0098
Aminosalicylates	103 (20.6%)	23 (16.0%)	80 (22.8%)	0.0896
Steroids	99 (19.8%)	24 (16.7%)	75 (21.4%)	0.2350
Enteral nutrition	69 (13.8%)	24 (16.7%)	45 (12.8%)	0.2618
Antibiotics	65 (13.0%)	30 (20.8%)	35 (10.0%)	0.0012
Anti-tuberculosis	27 (5.4%)	11 (7.6%)	16 (4.6%)	0.1705
Other agents^a^	115 (23.0%)	40 (27.8%)	75 (21.4%)	0.1251

^a^Other agents including traditional Chinese medicine, et al. *B1* non-stricturing non-penetrating, *B2* stricturing, *B3* penetrating, *BMI* body mass index, *L1* terminal ileum, *L2* colon, *L3* ileocolon, *L4* upper gastrointestinal tract

**Table 6:** Logistic model for each correlation factors to baseline perianal disease (has perianal disease vs. no perianal disease)

Variable	OR	95% CI for OR	Parameter estimates (standard error)	Corresponding 95% CI	*P* value
Age (years)	0.957	0.937, 0.977	−0.044 (0.011)+	−0.065, −0.023	<.001*
Sex (male vs. female)	1.072	0.701, 1.638	0.069 (0.216)	−0.355, 0.493	0.749
BMI (18.5 - < 24 vs. < 18.5 kg/m^2^)	1.082	0.632, 1.854	0.079 (0.275)	−0.459, 0.617	0.773
BMI (≥24 vs. < 18.5 kg/m^2^)	1.795	0.688, 4.683	0.585 (0.489)+	−0.373, 1.544	0.231
Nicotine use (former vs. Never)	0.209	0.048, 0.904	−1.564 (0.747)+	−3.028, −0.100	0.036*
Nicotine use (current vs. Never)	0.460	0.187, 1.136	−0.776 (0.461)+	−1.679, 0.127	0.092
Alcohol use (former vs. Never)	0.822	0.292, 2.313	−0.196 (0.528)	−1.231, 0.839	0.710
Alcohol use (current vs. Never)	0.389	0.113, 1.341	−0.943 (0.631)+	−2.180, 0.293	0.135
Time from disease onset to enrollment (months)	0.996	0.992, 1.000	−0.004 (0.002)+	−0.008, − 0.000	0.036*
Time from initial definite diagnosis to enrollment (months)	0.993	0.986, 1.000	−0.007 (0.003)+	−0.014, − 0.000	0.039*
Disease behavior (B2 vs. B1)	0.514	0.320, 0.827	−0.665 (0.243)+	−1.141, − 0.190	0.006*
Disease behavior (B3 vs. B1)	0.755	0.453, 1.256	−0.282 (0.260)+	−0.791, 0.228	0.279
Disease behavior (B3 vs. B2)	1.468	0.814, 2.646	0.384 (0.301)+	−0.206, 0.973	0.202
Disease location (L3 vs. L1)	3.052	1.834, 5.079	1.116 (0.260)+	0.607, 1.6252	<.001*
Disease location (L3 vs. L2)	2.485	1.320, 4.681	0.911 (0.323)+	0.277, 1.544	0.005*

“No perianal disease” is the reference level. +: standard error is not larger than parameter estimates. *: *P* < 0.05. *B1* non-stricturing non-penetrating, *B2* stricturing, *B3* penetrating, *BMI* body mass index, *CI* confidence interval, *L1* terminal ileum, *L2* colon, *L3* ileocolon, *OR* odds ratio

### Medical treatment and disease activity

At baseline, the mean (SD) CDAI score was 159.1 (91.45) and half of the patients (49.1%, 81/165) were in disease remission. Severe CDAI scores at baseline were reported in only 1.2% (2/165) of the patients. Patients enrolled in coastal medical centers had a higher proportion of moderate active disease (30.6% vs. 16.3%), and a smaller proportion of mild active disease (17.6% vs. 35.0%) than patients enrolled in inland centers (*p* = 0.0094).

At baseline, most of the patients (88.4%, 441/499) had a history of using any medicine for CD management. Less than 50% of patients were receiving immunomodulators (41.5%, *n* = 207; e.g., azathioprine) or anti-TNFα agents (32.9%, *n* = 164). About one-fifth of patients were on aminosalicylates (20.6%, *n* = 103) or steroids (19.8%, *n* = 99) (Table 1). Compared with inland centers, coastal centers had a higher use of anti-TNF agents (37.0% vs. 26.2%, *p* = 0.0123), enteral nutrition (20.5% vs. 3.1%, *p* < 0.0001), antibiotics (19.2% vs. 3.1%, p < 0.0001), anti-tuberculosis (8.4% vs. 0.5%, *p* < 0.0001) and other agents including traditional Chinese medicine (35.1% vs. 3.7%, *p* < 0.0001), but with a lower use of steroids (14.9% vs. 27.7%, *p* = 0.0005).

## Discussion

To our knowledge, this is one of the largest registry studies of CD in China till now, and the results reveal the disease characteristics, behaviors and treatment strategies of CD in China, including difference between inland and coastal medical centers. The results may be helpful to understand the current situation of CD in China. This registry study demonstrated that the number of male patients outweighed the number of female patients, which may reflect a higher prevalence of CD in men. Commonly reported symptoms of CD were abdominal pain, diarrhea, weight loss and fever. More than half of the patients had ileocolonic involvement, and half of the patients were of the non-stricturing non-penetrating behavior. Age, disease duration, B2 or B3 disease behavior and perianal disease were associated with CD-related surgery. This registry study also demonstrated that the diagnosis period was significantly shortened during the recent years in China.

Data of the present study demonstrated a male predominance in CD, with a male vs. female ratio of 2.30:1, which is consistent with the previous studies in Asian countries including data from Korea [7], Japan [17], India [18], Sri Lanka [19], Singapore [20] and others [21, 22]. In contrast, the incidence of CD in women is greater or equal to that in men in Western countries [23–25]. Thus far, no explanation is available for such discrepancies between Asian and Western populations, but this could be attributed to differences in genetic susceptibility and men having a greater possibility of seeking job opportunities in industrialized areas at a younger age, thus being exposed to potential environmental risk factors early in life. The peak incidence of CD observed in this study was in the fourth decade of life (32.3 years), consistent with previous reports from China [10, 26]. Studies in Western population have demonstrated a bimodal distribution in the age at onset of CD, with a peak occurring at 20–39 years and another peak at 50–79 years [27–30]. Studies in Asian populations have demonstrated either a less prominent second peak [7, 31, 32] or no second peak [33]. The exact reason for the bimodal distribution in the Western population is unclear, but it may be attributed to passive and active smoking in childhood and adulthood and sensitivities of different age groups to certain infectious factors [30, 34].

In terms of disease location, 56.2% patients had ileocolonic disease, whereas only 14.4% of the patients had colonic disease, which is consistent with the results from a single-center study from China [29]. Ileocolonic disease is also the most common CD phenotype in other Asian countries like Japan (65.8%) [35] and South Korea (77.7%) [7]. In Western countries, data on the disease location are relatively homogeneous largely with colonic, upper gastrointestinal and intestinal involvement. A population-based study from Hungary demonstrated that disease location for patients with CD was greatest in ileocolon (44.2%), followed by colon (35.6%), ileum (20.2%) and upper gastrointestinal region (2.4%) [36]. Disease behavior was clinically relevant as it is associated with the development of CD-related complications and need for surgery. At baseline, the disease behavior phenotypes (49.9% B1, 29.9% B2 and 20.2% B3) were concordant with a previous study from China and other studies from Asian countries [11, 33, 36]. Studies in western population have demonstrated that B1 was the most frequently observed behavior, with a frequency of nearly 80%. This difference might due to our registry patients who were not newly diagnosis CD patients, and the disease had already progressed to a more severe behavior. Accordingly, a larger proportion of patients change their behavior pattern to a more aggressive type (B2 and B3) after 5, 10 and 15 years of follow-up [37], and this may be attributed to increased smoking, disease location and clinical activity of the disease.

In concordance with other studies in the Asian population [36, 38, 39], abdominal pain, diarrhea and weight loss were the leading presenting symptoms of CD noted in the present study population. In the Western population, the incidence of diarrhea was the highest (79%), followed by abdominal pain (63%) and weight loss (21%) [40]. Certain extraintestinal manifestations such as oral ulcer, arthritis, skin lesions and uveitis often occur in patients with CD. The prevalence of extraintestinal manifestations was lower in the Asian population compared with the Western population (11% vs. 25–40%) [41–43]. Less frequent extraintestinal manifestations may be associated with relatively better prognosis among patients from Asian countries as compared with patients from Western countries.

Of the 499 patients, 23.8% (119/499) had history of CD-related surgery other than perianal disease surgery (15.8%). Older age at enrollment, longer disease duration, longer duration from disease onset to initial diagnosis and penetrating behavior were all risk factors related to CD surgery. A delayed diagnosis could result in missing the best timing for treatment, thus affecting the prognosis and increasing the risk of CD-related surgery [44]. Thus, diagnostic delay of CD is a challenge in several parts of the world. In Switzerland [45], the diagnostic delay was reported about 24 months. The analysis of the CD diagnosis period in the present registry revealed that the diagnostic period was significantly shortened in recent years. The current study found that patients with CD-related surgery history had a relatively lower presence of perianal disease. In the literature, patients with perianal disease carry a higher risk of surgery and this suggests a more severe disease course. [46] In the present study, patients with perianal disease also had a younger age at disease onset and a shorter duration from disease onset to enrollment, as well as a higher use of anti-TNF agents. These differences indicate that the presence of perianal disease may be shortened the diagnosis awareness and be helpful in differentiating from intestinal tuberculosis. A shorter diagnostic duration and a higher proportion of anti-TNF use might explain the negative association between perianal disease and bowel surgery.

In the present disease registry, immunomodulators were used frequently (41.5%) for the treatment of CD. A retrospective review in a population from East China demonstrated that 26.9% (61/227) patients had indications for immunomodulator use; however, such agents were prescribed to 34.4% of patients, of which, 37.5% received a subtherapeutic dose with no attempt of dose escalation [46]. The use of immunomodulators early in the course of the disease has been reported to be more effective in achieving clinical remission than conventional step-up therapy, but not in preventing relapse [47]. In Asia, anti-TNFs are used less frequently for the treatment of patients with CD. Sung et al. demonstrated that physicians in Asia favored anti-TNFs as a second-line therapy for CD [48]. A retrospective study from Korea, conducted from 1991 to 2007, reported that only 8.6% of patients with CD used infliximab [36]. Another study comparing the management of CD between Melbourne and Hong Kong demonstrated that a significantly higher percentage of patients were on anti-TNFs therapy in Melbourne than in Hong Kong (40% vs. 11%) [49]. Anti-TNFs are effective in inducing and maintaining clinical remission of CD [50, 51], and the use in the current study was higher (32.9%) than in other Asian countries. Thus, these results indicate that the update of new treatment paradigms was well adopted in China, especially in coastal medical centers. This multicenter study definitely helps inform a strategy for prevention and management programs for patients with CD in China by stratifying disease risk factors.

In the current study, because only baseline data were analyzed, disease progression and treatment effect could not be observed. Hence, further studies are warranted to describe the long-term disease progression. In addition, the small sample size of the current study may likely be interpreted as a selection bias. Patients were recruited from selected medical centers but not from national or regional registry. Since young patients < 18 years of age and women with pregnancy were excluded, the present study may not exactly reflect the profiles of the whole population. Adjuvant therapy such as enteral nutrition therapy, antibiotics and immunosuppressants may also affect the prognosis and disease behaviors.

## Conclusions

This multicenter, observational study was conducted in China to analyze the nationwide disease characteristics, clinical behavior and treatment strategies in patients with CD. This study indicated that the clinical features of CD in China were different from those in Western countries in terms of age, sex distribution, disease location, disease behavior, the prevalence of extra-intestinal manifestations and treatment practices. These findings have important implications for local resource allocation, healthcare planning and to further update the clinical guidelines for management of CD in China.

## Additional file

Additional file 1 : **Table S1.** List of participating centers; Full analysis set. **Table S2.** Subject dispositions; Screened analysis set. **Table S3.** Logistic model for each correlation factor to Crohn’s disease behavior (B1 vs. B2 + B3); Full analysis set. **Table S4.** Logistic model for each correlation factors to Crohn’s disease localization (L1 vs. L2 vs. L3 vs. L4); Full analysis set. (DOC 111 kb)

## Data Availability

All data generated or analyzed during this study are included in this published article and its supplementary information files.

## Abbreviations

B1: Non-stricturing non-penetrating
B2: Structuring
B3: Penetrating;
BMI: Body mass index
CD: Crohn’s disease
CDAI: Crohn’s Disease Active Index
CI: Confidence interval
FAS: Full analysis set
IBD: Inflammatory bowel disease
L1: Terminal ileum
L2: Colon
L3: Ileocolon
L4: Upper gastrointestinal tract
OR: Odds ratio
SD: Standard deviation
TNF: Tumor necrosis factor
