# Supplementary material for: Current diagnosis and management of Crohn’s disease in China: results from a multicenter prospective disease registry
Source: BMC Gastroenterol. 2019 Aug 16;19:145. doi: 10.1186/s12876-019-1057-2 (PMC6697932; doi:10.1186/s12876-019-1057-2)
Supplement: Supplementary file 1 — : Table S1. List of participating centers; Full analysis set. Table S2. Subject dispositions; Screened analysis set. Table S3. Logistic model for each correlation factor to Crohn’s disease behavior (B1 vs. B2 + B3); Full analysis set. Table S4. Logistic model for each correlation factors to Crohn’s disease localization (L1 vs. L2 vs. L3 vs. L4); Full analysis set. (DOC 111 kb) [file 12876_2019_1057_MOESM1_ESM.doc]

**Supplementary Data**

**Table S1.** List of participating centers; Full analysis set

| **Participating centers** | **City** | **Number of patients enrolled (N=499)** |
| --- | --- | --- |
| **North China** |  | 85 (17.0%) |
| Peking University First Hospital | Beijing | 5 (1.0%) |
| Army General Hospital | Beijing | 20 (4.0%) |
| Peking Union Medical College Hospital | Beijing | 60 (12.0%) |
| **East China** |  | 144 (28.9%) |
| Renji Hospital, Shanghai Jiao Tong University School of Medicine | Shanghai | 35 (7.0%) |
| Ruijin Hospital, Shanghai Jiao Tong University School of Medicine | Shanghai | 35 (7.0%) |
| Shanghai Tenth Peoples Hospital of Tongji University | Shanghai | 35 (7.0%) |
| Changhai Hospital, Second Military Medical University | Shanghai | 28 (5.6%) |
| Dongfang Hospital of Tongji University | Shanghai | 11 (2.2%) |
| **Central China** |  | 106 (21.2%) |
| The Secend Xiangya Hospital of Central South University | Changsha | 29 (5.8%) |
| Zhongnan Hospital of Wu Han University | Wuhan | 37 (7.4%) |
| The First Affiliated Hospital of Anhui Medical University | Hefei | 40 (8.0%) |
| **South China** |  | 164 (32.9%) |
| The First Affiliated Hospital, Sun Yat-sen University | Guangzhou | 75 (15.0%) |
| The Sixth Affiliated Hospital, Sun Yat-sen University | Guangzhou | 64 (12.8%) |
| Zhongshan Hospital, Xiamen University | Xiamen | 25 (5.0%) |

**Table S2.** Subject dispositions; Screened analysis set

|  | **Total** |
| --- | --- |
| **Analyzed for, N** | 504 |
| - Screening failure | 5 |
| - Full analysis set | 499 |
| **Completed, n (%)** | 421 (84.4%) |
| **Withdrawal, n (%)** | 78 (15.6%) |
| - Lost to follow-up | 60 (12.0%) |
| - Subject withdrew consent | 12 (2.4%) |
| - Death | 1 (0.2%) |
| - Others | 5 (1.0%) |

n: Total number of participants in a subset; N: total sample size.

**Table S3.** Logistic model for each correlation factor to Crohn’s disease behavior (B1 vs. B2+B3); Full analysis set

| **Variable** | **OR** | **95% CI for OR** | **Parameter estimates (SE)** | **Corresponding**  **95% CI** | **P value** |
| --- | --- | --- | --- | --- | --- |
| Age (years) | 1.003 | 0.988, 1.019 | 0.003 (0.008) | -0.012, 0.019 | 0.679 |
| Sex (male vs. female) | 1.354 | 0.921, 1.990 | 0.303 (0.196)+ | -0.082, 0.688 | 0.123 |
| BMI (18.5 - <24 vs. <18.5 kg/m^2^) | 0.908 | 0.563, 1.464 | -0.097 (0.244) | -0.574, 0.381 | 0.692 |
| BMI (≥24 vs. <18.5 kg/m^2^) | 0.622 | 0.246, 1.570 | -0.475 (0.473)+ | -1.402, 0.451 | 0.315 |
| Nicotine use (former vs. never) | 1.115 | 0.488, 2.552 | 0.109 (0.422) | -0.718, 0.937 | 0.796 |
| Nicotine use (current vs. never) | 2.454 | 1.152, 5.229 | 0.898 (0.386)+ | 0.141, 1.654 | 0.020* |
| Alcohol use (former vs. never) | 0.718 | 0.291, 1.771 | -0.331 (0.461) | -1.233, 0.572 | 0.472 |
| Alcohol use (current vs. never) | 1.268 | 0.530, 3.034 | 0.238 (0.445) | -0.635, 1.110 | 0.594 |
| Time from disease onset to enrollment (months) | 1.006 | 1.002, 1.009 | 0.006 (0.002)+ | 0.002, 0.009 | 0.002* |
| Time from initial definite diagnosis to enrollment (months) | 1.007 | 1.002, 1.013 | 0.007 (0.003)+ | 0.002, 0.013 | 0.009* |
| Baseline perianal disease (yes vs. no) | 1.251 | 0.848, 1.847 | 0.224 (0.199)+ | -0.165, 0.614 | 0.259 |

+: standard error is not larger than parameter estimates; *: P<0.05. B1: non-stricturing, non-penetrating; B2: Stricturing; B3: Penetrating; BMI: body mass index; CI: confidence interval; OR: odds ratio; SE: standard error.

**Table S4.** Logistic model for each correlation factors to Crohn’s disease localization (L1 vs. L2 vs. L3 vs. L4); Full analysis set

| **Factors** | **OR** | **95% CI for OR** | **Parameter estimates (SE)** | **Corresponding**  **95% CI** | **P value** |
| --- | --- | --- | --- | --- | --- |
| **Age (year)** |  |  |  |  |  |
| L2 vs. L1 | 0.987 | 0.964, 1.011 | -0.013 (0.012)+ | -0.036, 0.011 | 0.287 |
| L3 vs. L1 | 0.960 | 0.943, 0.978 | -0.041 (0.009)+ | -0.059, -0.022 | <0.001* |
| L4 vs. L1 | 0.993 | 0.937, 1.052 | -0.007 (0.030) | -0.065, 0.051 | 0.816 |
| **Sex (male vs. female)** |  |  |  |  |  |
| L2 vs. L1 | 0.691 | 0.371, 1.287 | -0.369 (0.317)+ | -0.991, 0.252 | 0.244 |
| L3 vs. L1 | 0.758 | 0.480, 1.197 | -0.277 (0.233)+ | -0.733, 0.179 | 0.234 |
| L4 vs. L1 | 2.469 | 0.294, 20.751 | 0.904 (1.086) | -1.225, 3.033 | 0.405 |
| **BMI (18.5 - <24 vs. <18.5 kg/m^2^)** |  |  |  |  |  |
| L2 vs. L1 | 0.404 | 0.192, 0.850 | -0.907 (0.380)+ | -1.651, -0.162 | 0.017* |
| L3 vs. L1 | 0.749 | 0.431, 1.299 | -0.290 (0.281)+ | -0.841, 0.262 | 0.303 |
| L4 vs. L1 | 1.043 | 0.219, 4.962 | 0.043 (0.796) | -1.517, 1.602 | 0.957 |
| **BMI (≥24 vs. <18.5 kg/m^2^)** |  |  |  |  |  |
| L2 vs. L1 | 0.258 | 0.052, 1.285 | -1.355 (0.819)+ | -2.960, 0.251 | 0.098 |
| L3 vs. L1 | 0.580 | 0.216, 1.555 | -0.545 (0.503)+ | -1.532, 0.441 | 0.279 |
| L4 vs. L1 | <0.001 | <0.001, >999.999 | -12.451 (583.715) | -1156.512, 1131.610 | 0.983 |
| **Nicotine use (former vs. never)** |  |  |  |  |  |
| L2 vs. L1 | 1.224 | 0.455, 3.294 | 0.202 (0.505) | -0.788, 1.192 | 0.689 |
| L3 vs. L1 | 0.205 | 0.071, 0.598 | -1.583 (0.545)+ | -2.651, -0.514 | 0.004* |
| L4 vs. L1 | <0.001 | <0.001, >999.999 | -12.631 (738.774) | -1460.602, 1435.339 | 0.986 |
| **Nicotine use (current vs. never)** |  |  |  |  |  |
| L2 vs. L1 | 1.526 | 0.579, 4.025 | 0.423 (0.495) | -0.547, 1.392 | 0.393 |
| L3 vs. L1 | 0.672 | 0.299, 1.514 | -0.397 (0.414) | -1.209, 0.415 | 0.338 |
| L4 vs. L1 | 3.891 | 0.674, 22.465 | 1.359 (0.895)+ | -0.395, 3.112 | 0.129 |
| **Alcohol use (former vs. never)** |  |  |  |  |  |
| L2 vs. L1 | 1.758 | 0.514, 6.010 | 0.564 (0.627) | -0.666, 1.793 | 0.369 |
| L3 vs. L1 | 0.694 | 0.235, 2.047 | -0.366 (0.552) | -1.448, 0.716 | 0.507 |
| L4 vs. L1 | 3.868 | 0.389, 38.513 | 1.353 (1.173)+ | -0.945, 3.651 | 0.249 |
| **Alcohol use (current vs. never)** |  |  |  |  |  |
| L2 vs. L1 | 1.582 | 0.523, 4.781 | 0.459 (0.564) | -0.647, 1.565 | 0.416 |
| L3 vs. L1 | 0.455 | 0.161, 1.286 | -0.787 (0.530)+ | -1.826, 0.252 | 0.138 |
| L4 vs. L1 | 2.900 | 0.302, 27.886 | 1.065 (1.155) | -1.199, 3.328 | 0.357 |
| **Time from disease onset to enrollment (months)** |  |  |  |  |  |
| L2 vs. L1 | 0.999 | 0.995, 1.004 | -0.001 (0.002) | -0.005, 0.004 | 0.830 |
| L3 vs. L1 | 0.999 | 0.996, 1.003 | -0.001 (0.002) | -0.004, 0.003 | 0.676 |
| L4 vs. L1 | 0.989 | 0.968, 1.010 | -0.011 (0.011)+ | -0.032, 0.010 | 0.290 |
| **Time from initial definite diagnosis to enrollment (months)** |  |  |  |  |  |
| L2 vs. L1 | 1.002 | 0.993, 1.010 | 0.002 (0.004) | -0.007, 0.010 | 0.701 |
| L3 vs. L1 | 1.005 | 0.999, 1.011 | 0.005 (0.003)+ | -0.001, 0.011 | 0.122 |
| L4 vs. L1 | 1.000 | 0.977, 1.024 | 0.000 (0.012) | -0.023, 0.023 | 0.992 |

+: Standard error is not larger than parameter estimates; *: P<0.05. BMI: body mass index; CI: confidence interval; L1: terminal ileum; L2: colon; L3: ileocolon; L4: upper gastrointestinal tract; OR: odds ratio; SE: standard error.
